# Colleague and patient appraisal of consultant psychiatrists and the effects of patient detention on appraisal scores

**DOI:** 10.1192/pb.bp.115.051334

**Published:** 2016-08

**Authors:** Miranda Heneghan, Robert Chaplin

**Affiliations:** 1 Royal College of Psychiatrists' Centre for Quality Improvement, London, UK

## Abstract

**Aims and method** This paper aims to review colleague and patient feedback from the 10-year period of the operation of the Royal College of Psychiatrists' 360-degree appraisal system, specifically: (1) examine the overall distribution of ratings; (2) examine the effect of working primarily with detained patients on patient feedback, represented by forensic psychiatrists; and (3) look for a relationship between colleague and patient ratings.

**Results** Data were analysed for 977 participating psychiatrists. Both colleagues and patients rated psychiatrists overall with high scores. Less than 1% were identified as low scorers, although there was no relationship between those identified by colleagues or patients. Colleague and patient feedback scores varied little between subspecialties including forensic consultants.

**Clinical implications** Psychiatrists in all subspecialties obtained high scores from colleagues and staff. Working with detained patients appeared to have little effect on patient ratings.

To receive revalidation from the General Medical Council (GMC),^1^ all doctors practising in the UK have to participate in annual appraisal. This has to include patient and colleague feedback (360-degree appraisal) at least every 5 years. Doctors of different specialties participate in various systems of appraisal involving colleague and patient feedback, including those organised by the Royal Colleges of general practitioners, physicians, surgeons and radiologists (under discussion but not yet finalised), and the GMC has produced staff and patient feedback tools.^2^ The Royal College of Psychiatrists has developed a system for collecting and analysing data from colleague and patient feedback and have validated questionnaires included in ACP 360,^3^ which has been shown to provide a reliable assessment.

Patient feedback ratings of a consultant psychiatrist's performance may be influenced by patient or consultant characteristics, or issues arising from the therapeutic alliance. Psychiatrists are unique among doctors as many of them treat patients who are detained and treated against their will. This study analyses the results of patient and colleague feedback received by consultant psychiatrists working in different subspecialties and specifically forensic psychiatrists as they work primarily with detained patients. It is hypothesised that consultants working in forensic psychiatry will have similar colleague ratings but inferior patient ratings of their performance when compared with consultant psychiatrists in other subspecialties.

## Method

Data were collected from the ACP 360 system administered by the Royal College of Psychiatrists' Centre for Quality Improvement (CCQI) received between September 2005 and December 2014. To obtain colleague feedback, consultant psychiatrists were asked to supply the email contacts of a range of 15 colleagues from their line manager, consultant psychiatrists, other clinical staff, junior doctors and secretarial, administration and management colleagues. Questionnaires were completed online on 46 items covering a variety of domains of consultant performance, including communication, availability, emotional intelligence, decision-making and relationships with patients and carers, relationships with psychiatrist peers and external agencies. A minimum of 25 patients who the consultant had had significant or recent contact with were sent a standard letter asking them to return a postal questionnaire, rating the consultant on 15 key areas of performance. Each item was rated on a scale of 1 (very low), 2 (low), 3 (moderately low), 4 (moderately high), 5 (high), 6 (excellent). Psychiatrists were grouped as low performing if their overall score was less than or equal to 3.

Data were analysed according to staff feedback and patient feedback separately by the types of subspecialty. To examine the effects of working with detained patients on the quality of their rating, feedback received from forensic psychiatrists was then compared with the other categories of subspecialties combined, referred to as ‘all other subspecialties’. This was because forensic psychiatrists work with detained patients and the other subspecialties only work with a minority of detained patients. Therefore, forensic psychiatrists were presumed to work with detained patients and all the other categories combined were presumed to work with patients who are not detained. It was hypothesised that forensic psychiatrists would receive lower ratings than their colleagues from patients but similar ratings from their colleagues. Data were entered onto the SPSS (version 21) database and statistical analysis was performed with non-parametric Mann–Whitney tests owing to the data being not normally distributed.

The ACP 360 system does not collect any further information about the participating psychiatrists such as demographics, so further analysis of the psychiatrists' individual characteristics and their influence on patient and colleague feedback is not possible.

## Results

Between September 2005 and December 2014, there have been 27 826 colleague and 20 543 patient ratings of 977 consultant psychiatrists. Although some psychiatrists will have participated in more than one round of feedback, this study is unable to identify how many have done so.

**Fig. 1:**
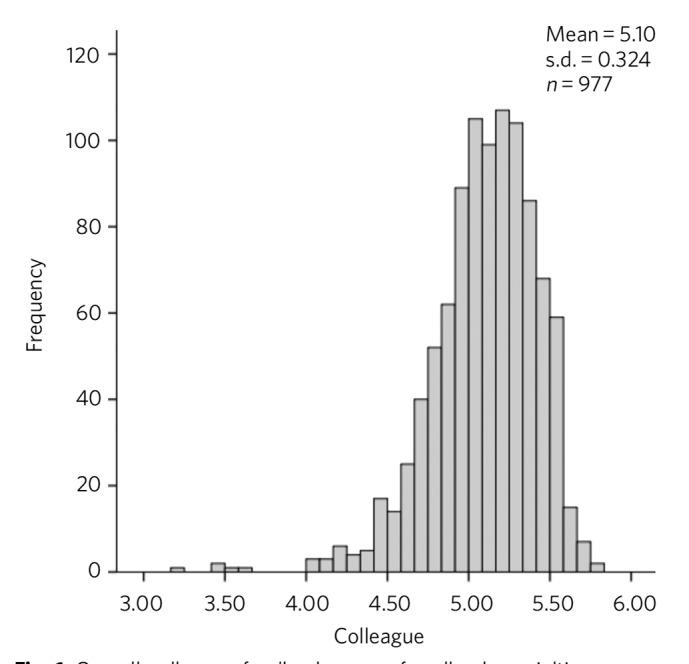
Overall colleague feedback scores for all subspecialties.

**Fig. 2:**
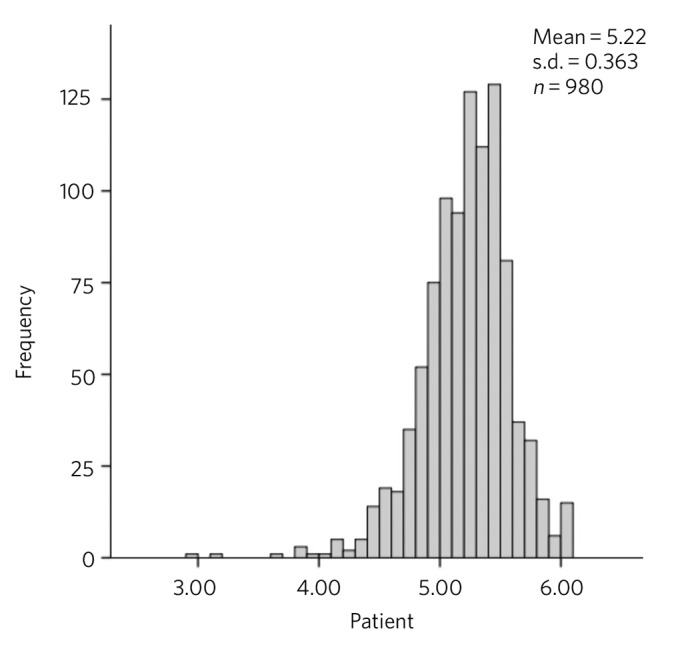
Overall patient feedback scores for all subspecialties.

**Fig. 3:**
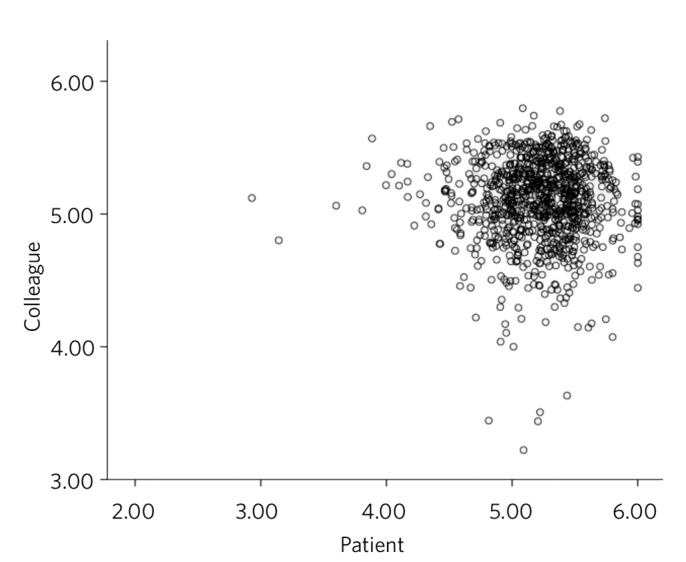
Correlation between colleague and patient feedback.

The distributions of overall scores rated by colleagues and patients are presented in Fig. 1 and Fig. 2 respectively. These ratings are clearly skewed towards the higher ratings and yield a mean overall score of 5.10 (s.d. = 0.293) from colleagues and 5.21 (s.d. = 0.362) from patients, where a maximum possible score was 6.0 for both colleagues and patients. Low performing psychiatrists were those whose mean scores were 4 or below (a score of below the highest scoring range). According to colleague feedback, 8 (0.8%) consultants scored an overall rating of below 4, whereas patient feedback identified 7 (0.7%) different consultants scoring below 4. There was no correlation between colleague and patient feedback (r = −0.21, *P* = 0.522) (Fig. 3).

Overall, there were 3891 returns of colleague feedback and 1765 patient returns from 133 forensic consultants (Table 1). Forensic consultants received an overall mean colleague assessment score of 5.04, which was slightly less and significantly lower than the overall score of ‘all other subspecialties’ of 5.11 (Mann–Whitney *U*-test, *P* = 0.02). The overall mean score for patient ratings of forensic psychiatrists was 5.23, not significantly different than that for ‘all other subspecialties’ (Mann–Whitney, *P* = 0.305).

**Table 1:** Colleague and patient rating scores for subspecialty of consultant psychiatrist

	Consultants *n*	Colleague scores	Patient scores
	
Specialty		Mean (range 1–6)	
Addictions	40	5.13	5.29

Child and adolescent psychiatry	6	5.15	5.16

Eating disorders	3	5.17	4.46

Forensic	133	5.04	5.24

General and community	501	5.11	5.20

Intellectual disability	2	4.72	5.23

Liaison	16	5.30	5.02

Old age	217	5.10	5.22

Perinatal	6	4.85	5.26

Psychotherapy	23	5.11	5.29

Rehabilitation and social	30	5.05	5.31

All except forensic	844	5.11^a^	5.21^b^

Total	977	5.11	5.21

a. Mann–Whitney *P* = 0.02 higher than forensic.

b. Mann–Whitney *P* = 0.30.

## Discussion

This study has shown that both colleagues and patients rate consultant psychiatrists highly, and that there is little variation in ratings of psychiatrists by either colleagues or patients between psychiatrists working in different subspecialties. High feedback rating scores from staff and patients are not a new finding. With regard to psychiatrists, the original pilot of the ACP 360^3^ yielded similar findings.

Multisource feedback tools in general obtain results from colleagues and patients that are highly skewed towards favourable assessments of performance of doctors' practising in a range of specialties.^4^ This study obtained multisource feedback on over 1000 practising doctors from a range of specialties including acute hospital care, mental health and primary care organisations, however, differences in rating scores achieved by doctors working in different specialties were not reported. Participating doctors were asked to provide lists of consecutive patients seen, so as to minimise the bias of obtaining responses from patients thought likely to respond positively. A study of multisource feedback on the performance of surgeons showed similar high ratings by patients (also consecutively selected) and colleagues, higher than the participants' self-ratings.^5^ However, these studies relied on the participants to provide their own lists of patients. To be more rigorous about the elimination of selection biases, Ramsey *et al*^6^ compared the results of staff respondents self-selected by the practising physicians in the USA with those randomly selected by their clinical directors and found no evidence of response bias.

This study identified a small number of psychiatrists rated as low performers: 0.8% by colleagues and 0.7% by patients. This suggests that the instrument is able to detect some individuals whose performance may be poor, although these are not the same individuals poorly rated by both patients and colleagues, a finding which differs from the positive correlation reported.^3^

With regard to the hypothesis that there would be no difference in rating of colleagues and lower ratings by patients of psychiatrists who worked primarily with detained patients (in this study represented by forensic consultants), there was no evidence to support this. This hypothesis was developed after staff working in the ACP 360 service started receiving feedback from psychiatrists believing that patient feedback would result in a negative assessment owing to having a poor therapeutic alliance resulting from working with detained in-patients. The relationship between detained status, the experience of coercion and the therapeutic alliance is complex and none of these factors have been studied specifically with regard to appraisal of a consultant psychiatrist's performance.

The effects of patients being detained under the Mental Health Act 1983 may have a potentially negative impact on the evaluation. For example, patients admitted to crisis houses rather than to acute mental health wards reported better therapeutic alliance and higher satisfaction with treatment.^7^ Further, there was a relationship between patients who perceived coercion (related to both informal and detained in-patient admissions) and a negative evaluation of the therapeutic alliance, suggesting the relationship between detention and poor therapeutic alliance is more complex than the legal situation of the patient.^8^ However, a study of in-patient admissions^9^ found that although detention in hospital predicted a poor therapeutic alliance, the perceived level of coercion on admission did not predict a negative therapeutic alliance. Finally, in a forensic in-patient setting, Donnelly *et al*^10^ found it possible for patients to reliably rate interpersonal trust and working alliance. These studies suggest that psychiatrists working with in-patients or detained patients may have a more negative rating from their patients.

The failure to support our hypothesis of forensic psychiatrists receiving lower patient ratings may come from a number of reasons. First, forensic psychiatrists may have long-term working relationships with patients (seeing the ‘usual’ doctor predicts positive ratings)^11^ and they may be perceived as serving a positive function, for example, rescuing a patient from prison. Second, detention in the community is now common and involves other specialties of psychiatrist, particularly general and community, so other specialties may be exercising coercion. It is of note that Campbell *et al*^12^ found that patients, but not colleagues, scored doctors from a mental health trust lower than doctors from acute and primary care trusts. This study when viewed alongside our study suggests that there may be factors general to all psychiatrists that influence patients' ratings that do not differ particularly across subspecialties.

It is known that patients seeing their ‘usual doctor’ are more likely to give positive feedback.^11^ There is less likelihood that low rates of forensic psychiatrists obtaining patient feedback are due to the same reasons, as longer-term therapeutic relationships are more likely in forensic in-patient settings. Other factors may include patients being unable to complete the feedback owing to lack of capacity. This may apply to psychiatrists working in forensic psychiatry and in psychiatric rehabilitation, although those working in intellectual disability distributed modified questionnaires which are not analysed here.

### Strengths and limitations

The ACP 360 is an instrument with robust psychometric properties which has been extensively field-tested. This study involved a large and representative group of psychiatrists from a variety of different subspecialties. However, owing to reasons of confidentiality, no further demographic data (e.g. age, gender, place of employment) were collected from the participants, therefore not permitting further analysis of psychiatrists' characteristics on performance. Furthermore, the categories of subspecialty are broadly defined, for example that of ‘general and community’ psychiatry could include a wide variety of posts including community mental health teams, acute in-patient admission wards and psychiatric intensive care units. Thus there may be variation in patient and indeed colleague rating within this broad category. However, given the narrowness of the patients' ratings and the probable high number of consultants working exclusively with in-patients contained within this group, a higher number of patients rating their consultants at the lower end of the scale would have been expected if detention status was to predicate lower patient ratings.

### Clinical implications

This study has two important implications. First, it suggests that the ACP 360 colleague and patient yield results skewed towards the higher ratings of performance in line with those received by doctors practising in other areas of medicine. Second, the appraisal tool does not wrongly discriminate against the performance of forensic psychiatrists who work almost exclusively with detained patients. It also is likely that patients treated by psychiatrists from other subspecialties may rate their psychiatrists in a manner which is not related to their detention. This study is not able to confirm this suggestion and further work into patient ratings of psychiatrists by individuals who have experienced detention or coercion is necessary.

The 360-degree appraisal is a helpful means of assessing psychiatrists' performance when used alongside other measures and psychiatrists need not fear negative ratings from patients when they work in services with high numbers of detained patients.
